# Dynamic Contrast Magnetic Resonance Imaging (DCE-MRI) and Diffusion Weighted MR Imaging (DWI) for Differentiation between Benign and Malignant Salivary Gland Tumors

**Published:** 2015-12-01

**Authors:** S. Assili, A. Fathi Kazerooni, L. Aghaghazvini, H.R. Saligheh Rad, J. Pirayesh Islamian

**Affiliations:** 1 Medical Physics Department, Faculty of Medicine, Tabriz University of Medical Sciences, Tabriz, Iran; 2 Quantitative MR Imaging and Spectroscopy Group, Research Center for Cellular and Molecular Imaging, Tehran University of Medical Sciences, Tehran, Iran; 3 Medical Physics and Biomedical Engineering Department, Tehran University of Medical Sciences, Tehran, Iran; 4 Department of Radiology, Shariati Hospital, Tehran University of Medical Sciences, Tehran, Iran; 5 Department of Radiology, Amir Alam Hospital, Tehran University of Medical Sciences, Tehran, Iran

**Keywords:** DCE-MRI, DWI, Salivary Gland Tumors, MRI

## Abstract

**Background:**

Salivary gland tumors form nearly 3% of head and neck tumors. Due to their large histological variety and vicinity to facial nerves, pre-operative diagnosis and differentiation of benign and malignant parotid tumors are a major challenge for radiologists.

**Objective:**

The majority of these tumors are benign; however, sometimes they tend to transform into a malignant form. Functional MRI techniques, namely dynamic contrast enhanced (DCE-) MRI and diffusion-weighted MRI (DWI) can indicate the characteristics of tumor tissue.

**Methods:**

DCE-MRI analysis is based on the parameters of time intensity curve (TIC) before and after contrast agent injection. This method has the potential to identify the angiogenesis of tumors. DWI analysis is performed according to diffusion of water molecules in a tissue for determination of the cellularity of tumors.

**Conclusion:**

According to the literature, these methods cannot be used individually to differentiate benign from malignant salivary gland tumors. An effective approach could be to combine the aforementioned methods to increase the accuracy of discrimination between different tumor types. The main objective of this study is to explore the application of DCE-MRI and DWI for assessment of salivary gland tumor types.

## Introduction

Salivary gland tumors form approximately 2-5% of head and neck tumors[1, 2]. They are located in sublingual, parotid and submandibular glands. According to world health organization (WHO), 54-79% of salivary gland tumors are benign and 21-64% of them are malignant[3]. Nearly 80% of salivary gland tumors occur in parotid glands. It should be noted that the majority of parotid tumors are benign (mostly pleomorphic adenoma) and a large number of minor salivary gland tumors are malignant[4]. Salivary gland tumors are very diverse in terms of histopathology and therefore, classification of these tumors has become a challenge for diagnosis, treatment and prognosis for surgeons and clinicians. Parotid glands are being divided into superficial and deep lobes by the facial nerve. Total parotidectomy is a common surgical procedure for malignant tumors, following which facial nerve may be lost, whereas for benign tumors, only a part of this nerve may be removed. Determining tumor location in relation to facial nerve is extremely important for surgeons which could be successfully accomplished by choosing an appropriate imaging modality[5].

Fine needle aspiration cytology (FNAC) and imaging are two ways to acquire accurate information from a tumor and for the clinician before any treatment planning and surgery. Even though FNAC is a common method, there are some limitations for the detection of malignant salivary gland tumors. For instance, due to diversification of malignant salivary gland tumors and small sample size, FNAC, as an invasive method, shows sampling errors. Therefore, preoperative imaging can help reduce these errors[6]. There are several imaging techniques such as ultrasound (US), computed tomography (CT), magnetic resonance imaging (MRI) and single-photon emission computed tomography (SPECT) for the evaluation of salivary gland tumors.

As the first step of diagnostic procedure, US may be applied for the detection of masses that are located in superficial parotid, submandibular and sublingual[7]. Schick et al achieved 72% sensitivity in determination of tumor types by using pulsed Doppler sonography[8]. For diagnosis of deep lobe masses, CT and MRI could be used. Arab et al compared the accuracy of SPECT with CT and MRI for diagnosis of salivary gland tumors. They showed that the accuracy of SPET, CT and MRI were 94%, 70-90%, 73-91%, respectively[9]. Rubello et al showed that fluorodeoxyglucose positron emission tomography (FDG PET), with or without CT, cannot distinguish between parotid tumor types[10].

Keyes JW et al reported that PET has 69% accuracy for classification of parotid tumors, which is poor in comparison with MRI[11]. The related literature indicates that CT, on top of its other limitations, is unable to prognosticate parotid tumors with acceptable accuracy[12, 13].

MRI has a good ability to differentiate various soft tissue types due to its superb spatial resolution. For example, T2-weighted MRI is a reliable technique to show whether tumors are benign or malignant. Furthermore, MRI is a non-invasive method without radiation hazards[14, 15]. Prades et al reported a sensitivity of 71% for diagnosing salivary glands malignancy using conventional MRI[16]. Nonetheless, detection of tumor location as well as tumor grading is difficult with conventional MRI[17].

In recent years, functional MR imaging techniques such as dynamic contrast enhanced (DCE) MRI and diffusion-weighted imaging (DWI) have significantly contributed to the diagnosis of head and neck tumors. It has been proposed that by combining apparent diffusion coefficient (ADC) map, derived from DW images with DCE-MRI, the diagnostic accuracy of  tumor types could be  improved[18-21]. The aim of this study is to review the existing literature on functional MR imaging modalities, namely DCE-MRI and DWI, for differential diagnosis of benign and malignant salivary gland tumors.

## Methods

### Information Source

A comprehensive search on Google Scholar and PubMed databases between 1990 and 2015 was performed applying these medical keywords: “dynamic contrast enhanced MRI” AND “diffusion weighted MRI” AND “salivary gland tumors”. The search was limited to human studies and English language papers. The references of preliminary and major studies were also reviewed to cover all related publications.

#### Criteria for Including Studies in this Review

This review focused on studies examining the clinical diagnostic value of MRI in salivary gland tumors. MR imaging should have been performed on 1.5 or 3T scanners using a head coil. Diagnostic accuracy was evaluated in comparison to gold standard of diagnosis, which was histopathological assessment results obtained after surgery for patients.

### Imaging Modality

In terms of morphology, salivary gland tumors are a diverse group of neoplasms, hence their clinical diagnosis has turned into a controversial problem[4, 22]. The clinical indications of malignant tumors could be painless and asymptomatic masses which are growing fast with partial paralysis of the facial nerve[23].

Clinical workup and diagnosis of parotid tumor consist of multiple steps. In the first step, physical examination is carried out by a physician for detection of palpable masses. Early diagnosis is essential for treatment planning; total parotodectomy is performed for malignant tumors while in benign patients local parotidectomy is done. Diagnostic radiology such as sialography, simple radiography, US, CT, PET and MRI might help in the diagnosis procedure of patients with ambiguous clinical appearances and physical symptoms. Conventional MRI is a routine clinical method that shows the extension of tumors to adjacent tissues. DCE-MRI is a non-ionizing diagnostic method, which is applied for tumor classification. This imaging modality is reproducible with high spatial resolution. Some studies have performed DCE-MRI of salivary gland tumors and obtained significant correlation between time intensity curves (TIC), analysis and histopathological findings. The concentration of contrast agent in the artery, the vascular surface area, permeability and other tumors features can be observed in DCE-MRI[24]. Thus, DCE-MRI can be exploited for assessment of tumor vascularization. DCE-MRI provides excellent soft tissue contrast compared to DCE-CT and DCE-US. DWI, as another functional imaging technique, can depict density and motion of water molecules as well as the effects of capillary perfusion in intracellular and extracellular space by calculating apparent diffusion coefficient (ADC). ADC values specify the amount of tumor cellularity. In what follows, the analysis of DCE-MRI, DWI and their combination are discussed for identification of different tumor types.

### Dynamic Contrast Enhanced (DCE-) MRI

DCE-MR imaging begins with injection of a paramagnetic contrast agent, which can induce signal enhancement through shortening of longitudinal relaxation time, T1, in nearby hydrogen atoms. A series of scans are acquired after contrast agent injection in a period of 1-3 minutes. Upon the entrance of contrast agent into a given tissue, signal intensity is changed in this region. The analysis of these changes provides useful diagnostic parameters. As an example, vessels produced by tumor angiogenesis appear fragile, leaky and with non-complete structures. DCE-MRI analysis can identify angiogenesis via comparing permeability and blood flow between the tumor and normal tissues.

Pharmacokinetic analysis is a DCE-MRI quantitative analysis method that was introduced by Larsson et al in 1990[25]. This model was later extended to Tofts generalized model. In brief, a simplified mathematical model is formed by two principal components: plasma volume through which the contrast agent passes and extracellular extravascular spaces. Considering signal intensity pattern, the rate of contrast agent administration through capillary membrane, plasma volume and extracellular extravascular space are obtained.

**Figure 1:**
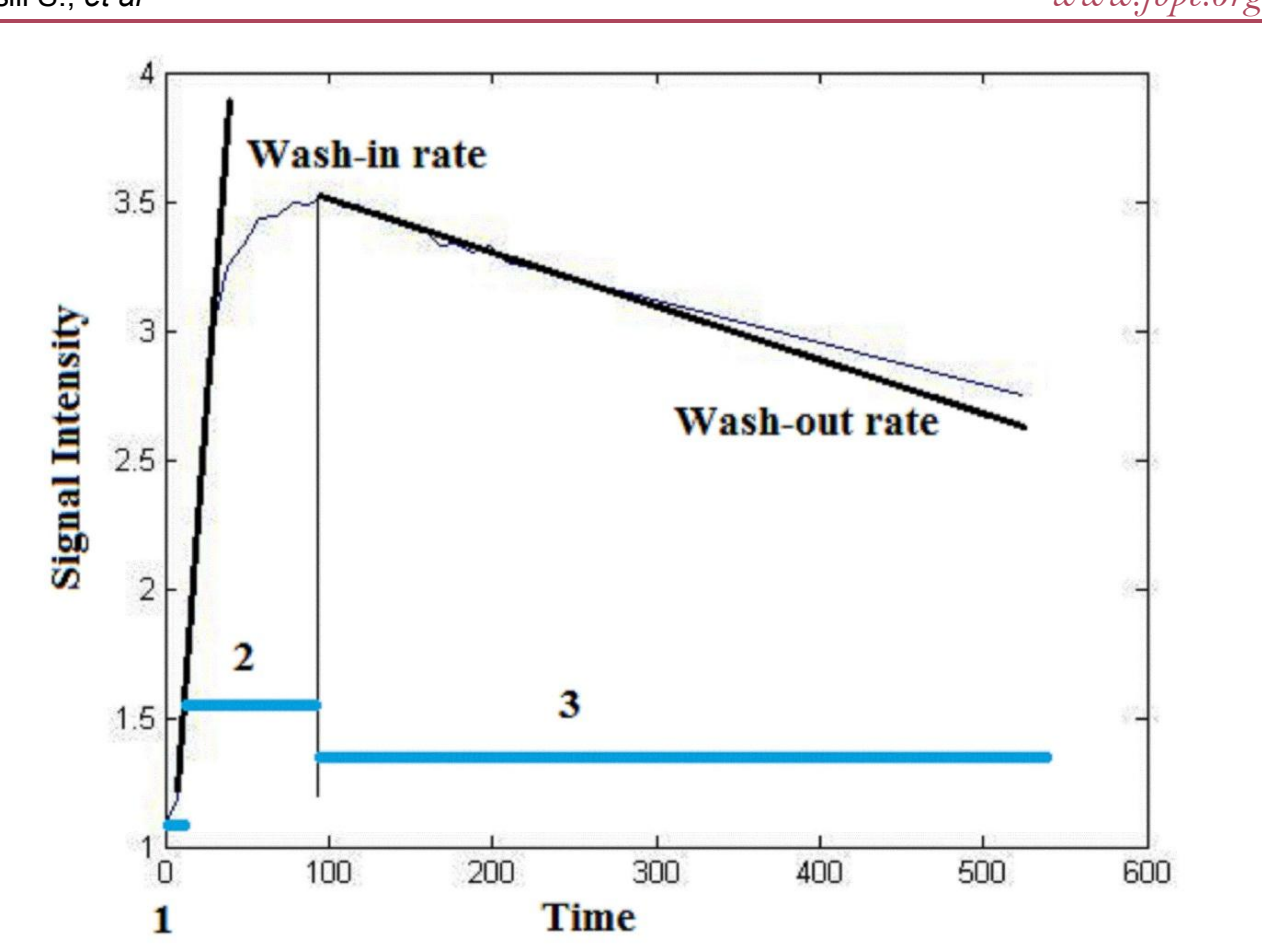
TIC Curve (three phase): before injection (1), wash-in (2), wash-out (3)

Time intensity curve (TIC) analysis is a method that uses the parameters derived from curves for classification of tumors. Three phases of TIC (before injection 1, washin 2, washout 3) are shown in Figure 1[26].

TIC patterns are different for benign and malignant tumors. For instance, in the case of malignant tumors, signal intensity curve in the first phase after contrast injection grows rapidly and in the next phase decreases gradually. In contrast, TIC curves for benign tumors have an increasing trend which can be used for classification of tumors[27].

The first DCE-MRI study on head and neck tumors was executed by Takashima et al in 1993. This study was performed on 79 head and neck lesions (69 patients). To evaluate signal intensity changes and increasing intensity patterns in this lesion, time-intensity curve for each lesion was plotted. Only time to peak (Tpeak) was used as a dynamic parameter for differentiation between benign and malignant tumors. After statistical analysis, the sensitivity of Tpeak showed no significant difference for tumor detection in comparison with non-contrast MRI. Based on TIC, lesions were divided into five groups: Tpeak <30 msec, 30<Tpeak<60, 60<Tpeak<120 and the curves with a gradual slope (without peak) and plateau curves. Takashima et al found that DCE-MRI could be useful for separation between pleomorphic adenoma and warthin tumors, but it could not distinguish between pleomorphic adenoma and adenoid cystic carcinoma or warthin tumors and other types of malignant tumors. There was some limitation in this study, including small number of malignant tumors[28].

Tsushima et al in 1994 characterized tumors of parotid gland and Parapharyngeal space using DCE-MRI[29].

Yabuuchi et al in 2000 performed a study on TIC analysis of salivary gland tumors. They compared TIC parameters and histopathologic findings in 29 patients with 33 salivary gland tumors. They also evaluated the relationship between tumor size and types and concluded that there was no meaningful statistical correlation between them. The slope, SI peak, Tpeak, enhancement ratio and washout ratio were obtained from TIC. These parameters were computed from the following equations:

WR= [(SImax - SI 5min)/ (SImax -SI pre)] × 100(%)   (1)

ER= (SImax - SIpre)/SIpre                  (2)

Slope = [(SIpeak -SIpre)/ (SIpre×Tpeak)] × 100   (3)

SI5: signal intensity 5 minutes after injection, SI pre: signal intensity before injection, SI max: maximum signal intensity

The statistical analysis showed a close relationship between T peak and micro vessel count and angiogenesis and WR also showed a close relationship with cellularity[18]. For instance, pleomorphic adenoma has long Tpeak due to small microvessel count and warthin tumor has short Tpeak due to large microvessel counts. This is similar to what obtained by Takashima et al[28]. Time intensity curves are categorized into 4 groups (A, B, C, and D) according to WR and Tpeak (Table 1)[18]. Yabuuchi et al found that by applying WR plus Tpeak, accurate detection of warthin tumors and malignant tumors would be possible.

**Table 1:** Classification of tumors by applying TIC parameters

A	Tpeak>120 sec, have a gradual enhancement (benign)
B	Tpeak=< 120sec (benign) WR>=30%, early enhancement and high wash-out,
C	Tpeak=<120sec (malignancy) WR<30%, early enhancement and low wash-out,
D	TIC was plateau (cystic lesion-benign) this categorized was performed before surgery.

In 2008, Eida et al evaluated a factor analysis of 2D dynamic MRI on salivary gland tumors. This study was performed on 36 salivary gland tumors (24 benign, 12 malignant). Eida et al applied pixel by pixel analysis due to effective power and accuracy of histopatholical discrimination. It should be noted that factor analysis in nuclear medicine was presented by Di Paola et al in 1982 for the first time for evaluation of organ structures[30]. Here, two parameters, (Tpeak and WR), were measured in the whole tumorous area. Using these parameters, lesions are classified into four groups: A) increasing curve without peak and contrast agent washout the period of 180 sec, B) increased initially and then washed out slowly, C) increased rapidly and quickly washout phase D ) flat curve[20]. The Tpeak as a physiological biomarker could predict cellularity amount, for example, short Tpeak indicates high cellularity[31, 32]. Eida et al believed that factor analysis of DCE-MRI methods could be an effective measure of tumor assessment before and after surgery. Decrease in cellularity after radiotherapy leads to changes in TIC in tumor area from B or C type to A or D type. In this research, by exploiting surface coils, spatial resolution increased compared to their previous study[33].

### Diffusion-Weighted Imaging (DWI)

Diffusion weighted imaging (DWI) depends on microscopic movement of water molecules, called Brownian motion. As a result of thermal excitation, water molecules move randomly, so DWI findings contain information about biological abnormality and cellularity at the initial stages of disease. Water diffusion is a three dimensional phenomenon. The mean of water diffusion rate can be measured in 3D which is called apparent diffusion coefficient (ADC)[34].

**Figure 2:**
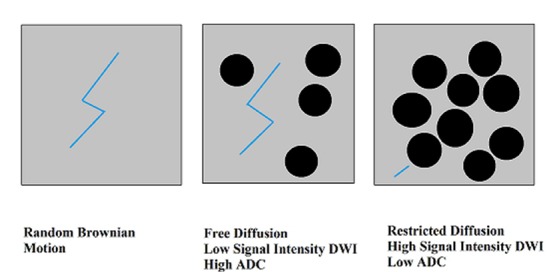
Water molecule diffusion, free diffusion and restricted diffusion

Diffusion of water in a tissue is influenced by several factors such as fluid viscosity, intracellular-extravascular membrane flow and structural direction that prevent or increase water mobility. Water molecules have random continuous movements due to continuous self-diffusion; each molecule goes through 20 µm in nearly a duration of 100 msec. The effect of this movement is enough to be measured with appropriate DWI pulse sequence by applying a strong pulse gradient echo. In isotropic diffusion process such as in tumors, diffusion is restricted in all directions. Since in high cellularity tumor tissues water molecules cannot move freely through extracellular space, such areas are seen as lesions with high signal intensity on DWI-MRI (Figure 2)[35].

**Figure 3:**
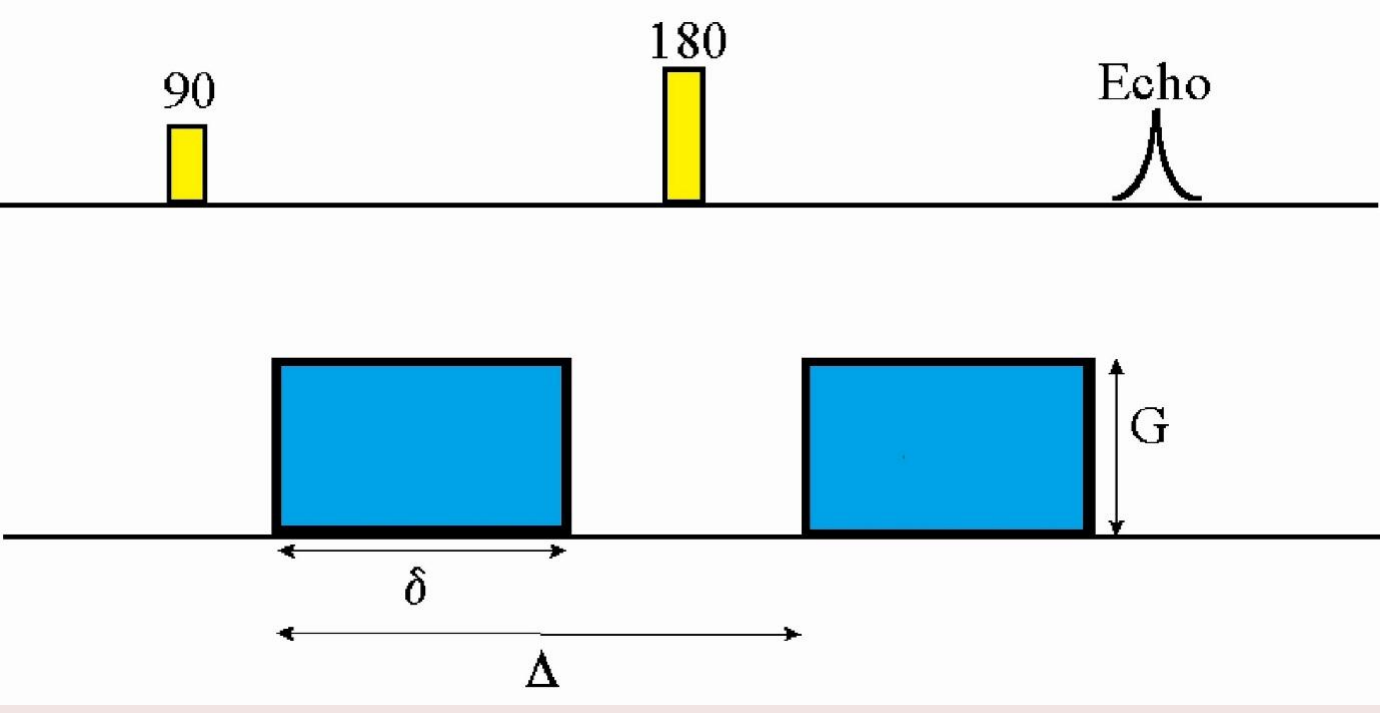
Diffusion weighted pulse sequence

At first, pulsed gradient spin-echo (PGSE) technique was introduced by Tanner and Stejskal. This gradient is sensitive to molecular movement. This pulse sequence describes two gradient echoes. The first gradient echo is applied for spin excitation and by using the second gradient echo, re-phasing happens (Figure 3)[36].The scattering phase could decrease signal exponentially[37]. Diffusion sensitivity is specified by the choice of b-values. The diffusion signal and the diffusion coefficient are calculated using the following equations:

b= (γ G δ) ^2^(Δ- δ/3)                          (4)

$$S=S_{0}e^{-{\left(\gamma G\delta \right)}^{2}\left(∆-\frac{\delta }{3}\right)D}=e^{-bD}\;(5)$$

G: gradient intensity

Δ: separation between applied gradient lobes.

δ: width of each gradient lobe

D: Diffusion coefficient

γ: gyromagnetic constant

S0: signal intensity without diffusion (on the T2-Weighted)

S: signal intensity of a voxel of tissue

The diffusion-weighted imaging is sensitive to histopathological changes. By correct b-value selection, obtaining functional and morphological information would be possible. The amount of b-value affects ADC, so that ADC measured from sequences with low b-value is significantly higher than this amount in sequences with high b-value[38].

Wang et al evaluated head and neck lesions using diffusion-weighted MR imaging. They calculated the mean ADC of all lesions; ADC mean for malignant tumors was 0.66 × 10^-3^mm^2^/sec, significantly it was smaller than carcinoma’s 1.13 × 10^-3^ mm^2^/sec (p < 0.001) and differ markedly for benign tumors, 1.56 × 10^-3^ mm^2^/sec (p=.002). The threshold for malignancy prediction was obtained that ADC means were smaller than 1.22 × 10^-3^ mm^2^/sec. the accuracy, sensitivity and specificity of this analysis are respectively 86%, 84% and 91%[39]. In another research, Heberman et al showed that DWI could be separated of 95 % of malignant parotid tumors[19].

In 2007, Eida et al assessed ADC maps for 31 parotid gland tumors (22 benign, and 9 malignant). Tumor types were identified by histopathologic methods, then, DW imaging was carried out on patients. ADC map was compared with histopathologic findings. They classified these regions into 4 groups. This categorizing was based on the ADC of this region related to whole tumor’s ADC:

Extremely Low ADC (ADC < 0.6 × 10^-3^] mm^2^/sec)

Low ADC: (0.6 × 10^-3^ mm^2^/sec< ADC < 1.2 × 10^-3^ mm^2^/sec)

Medium ADC: (1.2 × 10^-3^ mm^2^/sec< ADC < 1.8 × 10^-3^ mm^2^/sec)

High ADC: (1.8 × 10^-3^ mm^2^/sec < ADC)

In their investigation, benign tumors such as pleomorphic adenomas appeared homogenous mass on T1W images and heterogeneous mass on T2W images. The fast-growing tumor cells showed Mean ADC, high ADC indicated high cystic or myoxomatous regions[33].

Malignant tumors (e.g. adenocarcinoma and adenoid cystic carcinoma) could be observed as homogeneous masses on T1W and T2W images. Due to the existence of necrotic regions or cystic cellules in these tumors, they appeared inhomogeneous on ADC maps[33]. The malignant tumor was shown as a blue area with extremely low ADC and it has an apparent homogeneous on T1 and T2 images[33].

DWI, T1W and T2W and dynamic MRI were performed on these patients. ADC for warthin tumors was 0.96 × 10^-3^ mm^2^/sec and the amount of this for malignant tumors was reported 1.19 × 10^-3^mm^2^/sec; therefore, there was a threshold for differentiation between them (p<.01). They also investigated the relationship between b-value selection and ADC changes; for example, when applying low b-values, the amount of ADC is higher than when b-value is high, as perfusion can occur in the salivary gland region[33].

### Combination of DCE-MRI and DWI

In 2010, Eida et al studied the discrimination of benign and malignant parotid tumors by employing TIC and ADC-maps. The dynamic contrast enhanced and diffusion-weighted MRI were acquired on 70 patients with parotid tumors (52 benign and 18 malignant tumors). All of TICs were categorized into four groups based on the increment ratio (IR), time to peak (Tpeak) and washout rate (44): 

A (Benign tumors): IR<20%

B (Benign or Malignant tumors): Tpeak > 120 sec, IR ≥20 %

C (Malignant Tumors): Tpeak ≤ 120, WR < 30 %, IR ≥20 %

D (Warthin Tumors): Tpeak ≤ 120 sec, WR ≥ 30%, IR ≥20 %

Generally, when b-value is higher than 300 sec/mm2, it might contain perfusion. So, in this study, two b-values (500 and 1000sec/mm2) were used. These tumors were classified into four groups based on the mean ADC: extremely low ADC (ADC <0.6 ×10^-3^ mm^2^/sec), low ADC (0.6×10^-3^ mm^2^/sec ≤ ADC < 1.2×10^-3^ mm^2^/sec), medium ADC (1.2×10^-3^ mm^2^/sec < ADC ≤ 1.8×10^-3^ mm^2^/sec) and high ADC (ADC ≥ 1.8×10^-3^ mm^2^/sec).

According to TIC parameters analysis, Type A, type C and type D were diagnosed benign, malignant and warthin tumors, respectively[40]. Contrary to previous study performed by Yabuuchi et al, Type B has been reported malignant. Due to this reason, for more accurate differentiation of tumors, other parameters were needed[18]. The mean ADC combined with TIC parameters could greatly contribute to diagnosis. Based on mean ADC, areas with high (47±17%, 44±15%) and median ADC represented benign tumors and regions with low ADC (14±9 %, 28±14%) indicated malignant tumors. The best ADC threshold for differentiation between benign and malignant tumors was 40%. The sensitivity, specificity and accuracy for malignant tumors diagnosis were 86%, 100% and 97% (Table 2).

**Table 2:** The statistical analysis for diagnosis of the benign and malignant salivary gland tumors based on literature

	**year**	**Patient no**	**Imaging modality**	**Analysis**	**Sensitivity** **%**	**Specificity** **%**	**Accuracy** **%**
**Wang et al**	2001	97	DWI	ADC	84	91	86
**Yabuuchi et al**	2003	29	DCE-MRI	TIC	91	91	91
**Eida et al**	2007	31	DWI	ADC	89	100	97
**Eida et al**	2010	70	DWI, DCE-MRI	ADC	86	100	97
**Yabuuchi et al**	2008	47	DCE-MRI	TIC	71	86	83
**Yabuuchi et al**	2008	47	DCE-MRI, DWI	TIC , ADC	86	93	90

**Figure 4:**
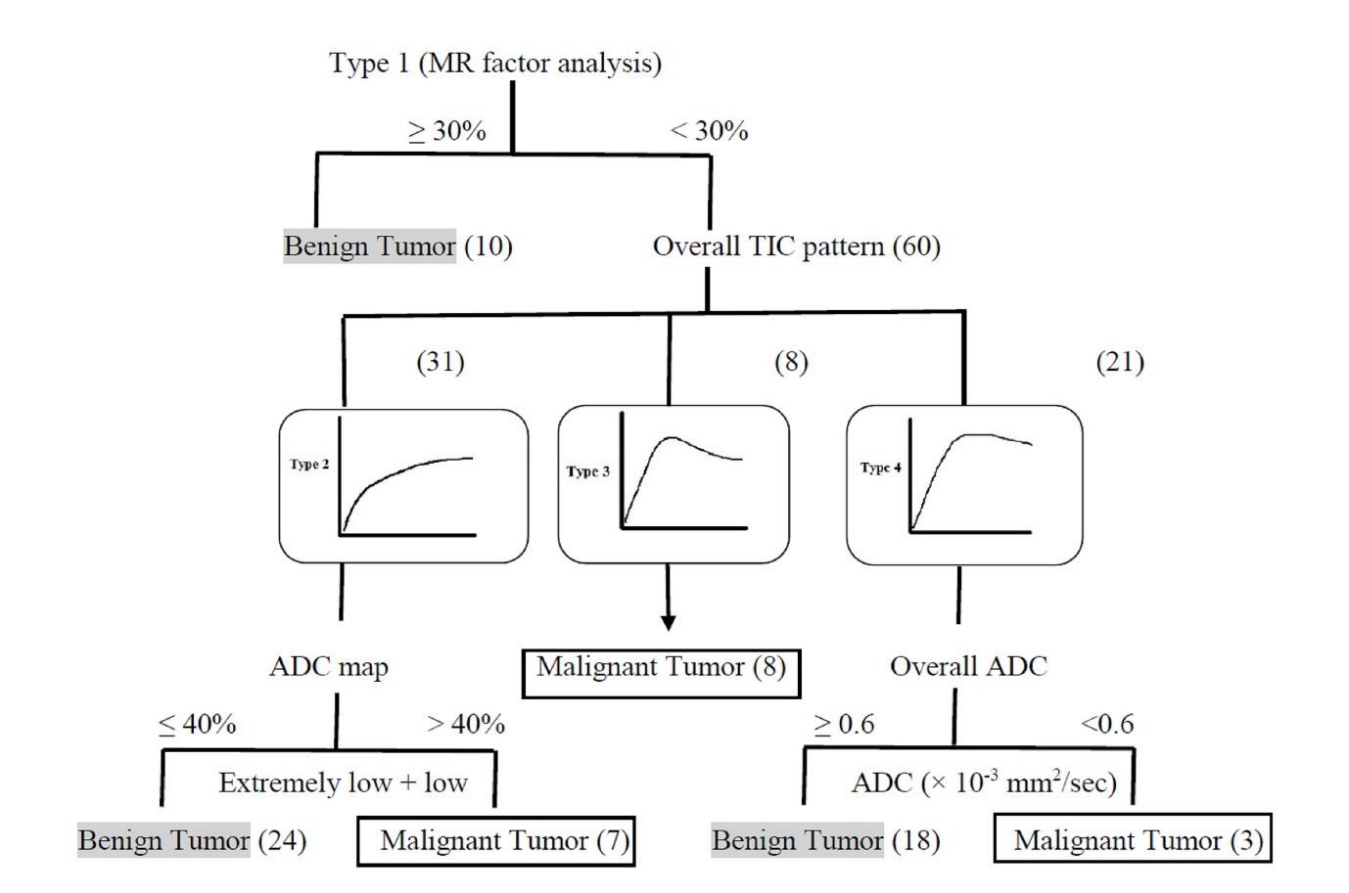
Decision tree for classification of parotid tumors by utilizing ADC and TIC analysis

Diagnosis diagram based on the combination of MRI multiple parameters is shown in Figure 4[40]. All tumors were classified into 2 groups, benign and malignant. This categorizing was performed according to DCE-MRI factor analysis and DWI analysis. They introduced new multi-parametric methods as a decision tree which helps better diagnosis of tumors, also previous studies problems such as overlapping parameters have been resolved by this method[33, 41].

In another study, Yabuuchi et al evaluated the diagnostic value of differentiation between benign and malignant tumors using DW and DCE-MR imaging. DWI and DCE-MRI were performed on 50 lesions (14 malignant and 36 benign), two weeks before surgery[21]. After imaging, for prevention of cystic area in analysis, regions of interest (ROIs) should be selected manually. In areas with non-uniform signal intensity, signal intensity of multiple regions was measured and areas with high signal intensity were selected. After calculating the average of signal intensity on ROIs, curve was plotted similar to previous study[18].TICs were divided into 4 types based on TIC parameters such as SI peak, WR and T-peak.

For ADC measurements, based on ROI of dynamic images, three regions were selected. Clearly, ADC values for pleomorphic adenomas were higher than that for carcinomas whereas ADC value for warthin tumors were lower than carcinomas’ (ADC for carcinomas) and ADC mean were divided into four groups (Table 3).

**Table 3:** The tumors classification based on ADC-Mean Value

**ADC-mean** ** ( ×10^-3^ mm^2^/sec) **	**Tumor Type**
1.92±0.36	Pleomorphic adenoma
0.83±0.16	warthin tumors
1.12±0.41	carcinoma
0.88±0.77	malignant lymphomas

After statistical analysis, ADC threshold between pleomorphic adenomas and carcinomas was identified 1.4 ×10^-3^ mm^2^/sec and between warthin tumors and carcinomas was 1.0 ×10^-3^ mm^2^/sec. Using TIC, sensitivity, specificity and accuracy were reported 71%, 86% and 83%, after adding DWI analysis to TIC results, sensitivity, specificity and accuracy were 86%, 93% and 90%. Therefore, multipara metric analysis can lead to early detection that can prevent unnecessary surgery and biopsy.

## Conclusion

Salivary gland tumors have a wide range in terms of histophatological findings. Moreover, lack of early detection causes tumor progression. For example, nearly 20 % of untreated pleomorphic adenomas could convert to malignant tumors. Thus, early detection and surgery helps to prevent recurrence [42]. Total Paroditectomy or local excision are common approaches for malignant and benign tumors. Hence, preoperative diagnosis has a great impact on undertaken treatment methods.

DCE-MRI and DWI are effective imaging methods in differentiating benign from malignant salivary gland tumors. The analysis of DCE-MR images can identify tumor angiogenesis and DWI is a useful method to determine the cellularity of tumors. As shown in different studies, each of these methods cannot be applied individually for reliable differentiation of tumor types. For instance, in studies performed by Yabuuchi et al, based on TIC analysis, type 2 tumors were diagnosed as benign tumors, whereas Eida et al showed that tumors with type 2 can be either benign or malignant[18]. Eida et al demonstrated that by using TIC analysis plus ADC map, could enhance diagnosis. Such stepwise methods could be effective in determination of tumor subtypes[40]. Combination of DCE-MRI and DWI may be effective for separation of salivary gland tumors and could provide reliable information to increase the diagnostic accuracy for physicians.
